# Employing Cellulose Nanofiber-Based Hydrogels for Burn Dressing

**DOI:** 10.3390/polym14061207

**Published:** 2022-03-17

**Authors:** Aliakbar Tofangchi Kalle Basti, Mehdi Jonoobi, Sima Sepahvand, Alireza Ashori, Valentina Siracusa, Davood Rabie, Tizazu H. Mekonnen, Fatemeh Naeijian

**Affiliations:** 1 Department of Wood and Paper Sciences and Technology, Faculty of Natural Resources, University of Tehran, Karaj 77871-31587, Iran; tofangchiali840@gmail.com (A.T.K.B.); sepahvand.s@ut.ac.ir (S.S.); 2 Department of Chemical Engineering, University of Waterloo, Waterloo, ON N2L 3G1, Canada; tizazu.mekonnen@uwaterloo.ca; 3 Department of Chemical Technologies, Iranian Research Organization for Science and Technology (IROST), Tehran 33531-36846, Iran; 4 Department of Chemical Science, University of Catania, Viale A. Doria 6, 95125 Catania, Italy; 5 Director Scientific Committee of Iran National Council of Furniture, Tehran 15147-35418, Iran; davoodrabie@gmail.com; 6 Department of Biorefinery Engineering, Faculty of New Technologies and Energy Engineering, Shahid Beheshti University, Zirab 19839-69411, Iran; fatemeh.naijian@gmail.com

**Keywords:** burn dressing, cellulose nanofibers, hydrogel, cross-linker

## Abstract

The aim of this research was to fabricate a burn dressing in the form of hydrogel films constructed with cellulose nanofibers (CNF) that has pain-relieving properties, in addition to wound healing. In this study, the hydrogels were prepared in the form of film. For this, CNF at weight ratios of 1, 2, and 3 wt.%, 1 wt.% of hydroxyethyl cellulose (HEC), and citric acid (CA) crosslinker with 10 and 20 wt.% were used. FE-SEM analysis showed that the structure of the CNF was preserved after hydrogel preparation. Cationization of CNF by C_6_H_14_NOCl was confirmed by FTIR spectroscopy. The drug release analysis results showed a linear relationship between the amount of absorption and the concentration of the drug. The MTT test (assay protocol for cell viability and proliferation) showed the high effectiveness of cationization of CNF and confirmed the non-toxicity of the resulting hydrogels.

## 1. Introduction

Hydrogels are a group of polymeric materials made up of three-dimensional (3D) network structures with physical or chemical cross-links that have unique properties, such as a hydrophilic nature, high sensitivity to the physiological environment, good water absorption power, and optimal flexibility [1]. The water absorption capacity of hydrogels arises from the hydrophilic functional groups in their polymer chains (including hydroxyl, carboxyl, amine, and sulfonate groups), and their resistance to dissolution is due to cross-connections between network chains [2]. One application of hydrogels is their use as a dressing (wound and burn). In film or amorphous form, hydrogels’ hydrophilic nature accelerates the healing process [3]. Burn dressings can be divided into traditional and modern dressings. Traditional dressings usually have a non-stick coating on the wound contact area side that acts as an absorbent layer around the wound [4]. A disadvantage of conventional skin tissue repair materials, such as gauze, is their strong permeability, which leads the repair material to firmly adhere to the surface of the dry wound, resulting in new damage when the dressing is removed. Modern wound dressings developed to facilitate wound function focus more on protecting the wound from dehydration and wound healing. Such modern dressings are available in film, foam, hydrogel, and hydrocolloid form. These dressings act as a barrier against bacteria that can penetrate the wound environment [5,6]. Hydrogels can be used as amorphous gels or in the form of films. Polymer compounds physically trap water by creating a cross-link to make a hydrogel film. Thus, the hydrogel dressing needs to be frequently replaced when applied to the wound in the form of a gel, and a secondary coating, such as gauze, should be used [7]. Semi-permeable polymer film hydrogel dressings control water vapor transfer and retain moisture; these types of dressings are very effective for treating and healing burn patients in the shortest time possible [7,8].

Hydrogels as a burn dressing have many benefits, including the absorption and retention of wound secretions, which increase fibroblast proliferation and keratinocyte migration. These two processes are essential for epithelialization and wound healing. In addition, the small size of the hydrogel microstructure protects the burn from infection and prevents microorganisms from reaching the wound. The hydrogel structure allows the transfer of bioactive molecules, such as antibiotics and drugs, to the burn center. These molecules can enter the hydrogel networks during the dressing process. In general, hydrogels have a high ability to absorb wound secretions, accelerate burn healing, reduce pain and inflammation, are inexpensive, and stable in severe temperature fluctuations. Most importantly, they do not stick to the burn site and cause pain after detachment from the wound surface. In addition, they are suitable for tissue regeneration due to their biocompatibility, transparency, and biodegradability [9,10]. The conventional wound dressings, including natural or synthetic bandages and cotton wool, have an extended treatment period and may even adhere to dried wound surfaces. Therefore, hydrogel-based wound dressing has received special attention due to its multifaceted properties, such as cooling sensation, humid environment, and absorption of wound secretions [11].

Wounds are defined as a disorder in the integrity of the epithelial lining of the skin or mucosa caused by physical and thermal damage [4]. In fact, injuries on the skin are generally considered wounds. Wounds are classified into acute and chronic. An acute wound is usually superficial and is a type of skin damage that occurs suddenly as a result of an accident or surgery. Depending on its depth and size in the epidermis of the skin or dermis layers, it can heal in two to three months. However, chronic wounds are life threatening because they do not heal in time. Chronic wounds include burns, decubitus ulcers, infections, leg ulcers, etc. [12,13]. Burn injury reduces the function of the immune system and increases susceptibility to infection. In addition, the greater the depth of the burn, the higher the mortality rate [14].

Unfortunately, hydrogels suffer from poor mechanical stability in their swollen state. This problem can be resolved by using a “composite or hybrid hydrogel membrane” system made up of more than one polymer in the dressing configuration [8]. In a cross-linking method, chemical or physical interactions between polymer chains stabilize the hydrogel. Cross-linking allows the mesh structure of hydrogels to retain large amounts of absorbed water. Physical and radiation cross-linking are favored over chemical cross-linking to form a hydrogel, especially for biomedical applications [8]. Chemical and physical cross-linking between macromolecular chains allows hydrophilic polymers to adsorb and desorb water without dissolving the polymer. The network structure of the polymer determines the swelling capacity, the compatibility of the polymer solvent, the amount of cross-linking, and the ionic reaction. The controlled swelling of the cellulose nanofiber (CNF) 3D network provides an unparalleled opportunity for this new material to be used in dressing management [15].

Medical applications of CNF have expanded dramatically in recent years; this includes creating suitable hydrogels for wound dressing, wound healing, and infection prevention [16]. The extraordinary physical and biological properties of CNF, particularly its naturalness, great abundance, biocompatibility, biodegradability, stability, non-toxicity, low price, and outstanding mechanical properties, make these materials attractive for medical applications. These applications include implants, tissue engineering scaffolds, drug release agents, wound healing, and cardiovascular applications [17,18,19,20]. One advantage of CNF is that its surface can be easily modified due to the abundant -OH structural moieties. Surface modification can increase material performance in various ways [21,22,23]. For example, cationic CNF created with quaternary ammonium compounds produce materials with antimicrobial properties, which have many potential applications in the biomedical and pharmaceutical fields. Although pristine CNF can be used in wound dressing, it does not have intrinsic antibacterial properties. Therefore, CNF needs to be activated with an antiseptic drug [24]. Many studies on wound healing have shown that CNF is an excellent candidate for wound dressing materials design attributed to the high surface area for contact and more exposed hydroxyl groups than cellulose fibers [16,24].

Liu et al. (2018) examined hydrogels created from cellulose nanofibers, gelatin, and aminated silver nanoparticles for wound dressing applications. They concluded that the manufactured hydrogels have good mechanical properties and good antibacterial and hemostatic properties. In addition, they have a proper function in balancing the wound bed and improving wound healing [11]. Portela et al. (2019) discussed the use of bacterial cellulose as a biopolymer to accelerate wound healing. They showed that bacterial cellulose, due to its high purity and porosity, permeability to liquid and gases, high water absorption capacity, high mechanical strength, antibacterial properties, biocompatibility, and potential for drug delivery, can be used as effective wound dressing material [25]. Abazari et al. (2021) provided a complete description and analysis of wound-healing structures and dressings made from cellulose-based biomaterials. They showed that cellulose-based biomaterials have promising potential as dressings and wound-healing agents. In addition, they can be integrated with various bioactive agents. In general, cellulose-based biomaterials can form effective structures for wound dressing and healing [26].

Although CNF is a unique biomass material with superior mechanical and physical properties, few studies have been conducted on the use of CNF in the preparation of burn repair hydrogels or controlled drug loading and release. The objective of this research is to fabricate a CNF hydrogel in the form of a film or sheet and evaluate it in a topical dermal drug delivery system. Compared to traditional wound dressings on the market, this hydrogel could have the advantage of a gradual and controlled drug release. Thus, the primary aim of this research is to prepare a hydrogel that has restorative, pain-relieving, and antiseptic properties. Moreover, the constructed hydrogel’s water absorption capacity and mechanical strength to provide genuine benefits are evaluated.

## 2. Materials and Methods

### 2.1. Materials

In this study, cellulose nanofibers (CNF) from the Weidmann Company (Rapperswil, Switzerland) were obtained by grinding a disk with an average fiber diameter of 38 nm. Hydroxyethyl cellulose (HEC) with a molecular weight of 250,000 g/mol and a viscosity of 80–125 cps was purchased from Sigma Aldrich Germany. Citric acid (CA) used as a cross-linker was purchased as a powder from Sigma Aldrich Germany. Additionally, the silver nitrate (AgNO_3_), used for its antiseptic properties in wound dressing, was supplied by Sigma Aldrich Germany. It is recognized that AgNO_3_ has low toxicity and provides burn repair in burn dressing, pain relief, rapid release, and control of excess tissue [4,27]. All the chemicals and materials used in this study are of the highest purity, and all the solutions were prepared with the use of distilled water.

### 2.2. Hydrogel Fabrication

**Figure 1:**
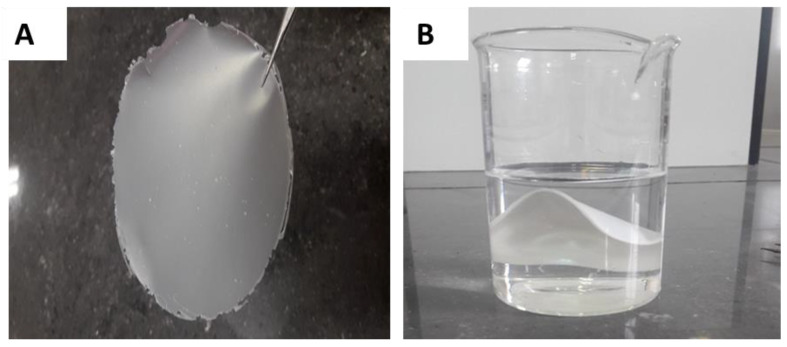
Image of a hydrogel with a ratio of CNF/HEC (3:1 wt.%) and CA 20 wt.% ((**A**): in the dry sample and (**B**) in the wet sample).

Aqueous suspensions of CNF at three different concentrations (1, 2, and 3 wt.%) were prepared in distilled water. Since the CNF are suspended in water, they have a high tendency toward intramolecular interaction and agglomeration. To resolve this challenge, HEC was incorporated in the CNF suspensions to reduce intramolecular and intermolecular interactions among CNF chains and increase intermolecular bonds between CNF and HEC. For this, 1 wt.% CHEC solution was prepared by continuously stirring the HEC powder in water at room temperature for 2 h until the solution was clear and uniform, and the viscosity increased. To prepare the CNF/HEC mixtures, the HEC solution was added to each of the prepared CNF suspensions. The CNF/HEC mixtures were then stirred at room temperature for 6 h, and subsequently, 10 and 20 wt.% with respect to the dry weight of CNF of the CA cross-linker was added (Table 1). Since CA must lose water to make cross-links, the samples were placed in an oven at 30 °C for 24 h to evaporate the excess water. They were then transferred to an oven at 80 °C for 7 h to generate the cross-links. The constructed hydrogel was placed into a vacuum oven at 50 °C and relative humidity of 50% for 24 h to complete the cross-linking process (Figure 1).

### 2.3. Characterizations

#### 2.3.1. Attenuated Total Reflectance-Fourier Transform Infrared Spectroscopy (ATR-FTIR)

The functional groups, bonds between chemical compounds in hydrogels, and cross-links were observed using ATR-FTIR spectrometry with a Perkin Elmer Frontier TM spectrometer in a spectral range from 400 to 4000 cm^−1^ and a scanning resolution of 4 cm^−1^ (32 scans for each sample).

#### 2.3.2. Gel Fraction

In order to obtain the gel fraction (cross-linking efficiency), the sample was placed in a vacuum oven at 50 °C for 5 h, and then, the dry weight of the sample was first recorded. Then, each sample was placed in a separate container in 200 mL of distilled water at room temperature for a week. During this week, the water was changed 3 times a day. One week later, the hydrogels were placed in a vacuum oven at 50 °C for 12 h. During that week, the water of the samples was changed 3 times a day. Then, the dry weight of the sample was measured before (M) and after (M’) washing, and the gel fraction (cross-linking efficiency) was determined using Equation (1) [28]:

(1) $$\mathrm{Gel}\;\mathrm{Fraction}\;(\%)=\frac{M^{\prime }}{M}\times \;100$$

#### 2.3.3. Field Emission Scanning Electron Microscope (FE-SEM)

The hydrogels morphology was examined by a field emission scanning electron microscope (FE-SEM), TESCAN MIRA2 (TESCAN, Brno, Czech Republic) with a voltage of 7 kW. The samples were fixed on copper plates by conductive double-sided adhesive tapes and coated with 15 nm thick gold.

#### 2.3.4. Rheological Characterization

A flow behavior test was performed to confirm cross-linking and investigate the elastic modulus of the hydrogels in a completely swollen state. A HAAKE RS 6000 rheometer (Thermo Fischer Scientific, Waltham, MA, USA) was used to measure the rheological properties of the hydrogels with a thickness of 2 mm, a diameter of 15 mm, and a temperature of 32 °C. A variable frequency test in the linear viscoelastic region with a constant strain value was determined for each sample separately, and the modulus of elasticity or storage modulus (G’) and viscous modulus or loss modulus (G”) were determined with variable frequency in the range of 0.1–8 Hz.

#### 2.3.5. Drug Uploading

The dried samples were immersed in distilled water, and the hydrogels were weighted again. Then, the moisture content was brought to 50% of its moisture absorption capacity [29]. The hydrogel samples were then immersed into a 10% solution of silver nitrate with pH = 7.4 at a temperature of 30 °C under stirring (200 rpm), for 24 h. After absorption of the silver nitrate, the samples were placed in an oven at 40 °C for 16 h.

The dried samples were weighed and then immersed in distilled water and weighted again. Then, the moisture content was adjusted to 50% of its moisture absorption capacity [29]. The hydrogel samples were loaded with a 10% solution of silver nitrate with pH = 7.4 at a temperature of 30 °C under stirring (200 rpm), for 24 h. After absorbing the silver nitrate drug, the samples were placed in an oven at 40 °C for 16 h. Drug loading was determined according to the concentration of the solution before and after loading.

#### 2.3.6. Drug Release Mechanism

To determine drug release (at room temperature, 32 °C), 200 mL of phosphate-buffered saline (PBS) solution with pH = 7.4 was employed as a release medium. For this purpose, the loaded hydrogel was taken from the loading solution and transferred to the PBS release medium. After that, a stirrer with a rotation speed of 200 rpm was used for 12 and 24 h, and 1 mL of the release medium was sampled to measure the concentration of the drug. In order to keep the volume of the release solution constant, 0.1 normal PBS solution was added to the release medium at the same rate after each sampling (1 mL). Using ultraviolet-visible spectroscopy (Cary 60 UV-Vis, company Agilent Technologies, Santa Clara, CA 95051, USA), the amount of silver ions in the medium was determined after each sampling. Since the data extracted from this device only show the amount of absorption at a certain wavelength, a calibration curve was needed to achieve the relationship between the concentration and the adsorption rate (230 nm). Therefore, the adsorption amount was measured in solutions with a certain concentration of the drug in the range of concentrations used in the loading by the UV-Vis. The relationship between the concentration and the amount of adsorption was determined by fitting the regression line and obtaining the formula of the fitted line, and the concentration of each test sample was obtained by numbering the amount of adsorption in the release test samples. Since replacing the amount of the test sample emerging from the solution with the PBS solution changes the concentration of the release medium, the actual cumulative concentration of the released drug at each time point (*C_real_*) was calculated using Equation (2):

(2) $$C_{real}=C_{t}+\frac{V_{t}}{V_{r}}\sum_{i=1}^{i=t-1}C_{i}\;$$

where *C_t_*, *V_t_*, and *V_r_* are the apparent cumulative concentration of the drug released at time *t*, the amount of substance taken out each time, and the total amount of the released solution, respectively.

The cumulative amount of drug released at time *t* (*M_t_*) is obtained by multiplying the concentration of the released test specimens by the amount of 0.1 normal PBS release solution according to Equation (3):

(3) $$M_{t}=V_{r}C_{t}+V_{t}\sum_{i=1}^{i=t-1}C_{i}\;$$

Finally, according to Equations (2) and (3), the result is the cumulative percentage of drug release (*Z*) over time which *M_∞_* and *M_t_* are the cumulative amounts of drug released at the infinite time and the amount of drug in the hydrogel after loading, respectively. This was calculated using Equation (4) [30]:

(4) $$Z=\frac{M_{t}}{M_{\infty }}\times 100=\frac{V_{r}C_{t}+V_{t\;}\sum_{i=0}^{i=t-1}C_{i}}{M_{\infty }}\times 100$$

The Peppas-Ringer quasi-experimental equation (Equation (5)) was used to model the release data [31]:

(5) $$\frac{M_{t}}{M_{\infty }}=K^{n}$$

The percentage of cumulative release logarithms [log (*M_t_*/*M_∞_*)∗100] was drawn in the range of 5 to 60% compared to the logarithm of the release time [log (*t*)]. A linear regression line was fitted to the data, and the slope (*n* = diffusion power) was used as an indicator for the release mechanism. The release mechanism of the exponential coefficient (*n*) is described in Equation (6):

(6) $$\frac{M_{t}}{M_{\infty }}=Kt^{n}$$

The values of *n* equal to 0.45 and 0.5 in the cylindrical and plate-shape samples indicate the release mechanism as Fick’s infiltration. In fact, Fick’s release is the same as Higuchi’s release model, where release control is performed through Fick’s release. In this case, the release of the drug is controlled by infiltration, whereas a release mechanism with exponential coefficients of 0.89 and 1 in cylindrical and plate-shape samples, respectively, is affected by the control system through swelling.

#### 2.3.7. Biocompatibility of Hydrogels

The biocompatibility and non-toxicity of the hydrogels were evaluated according to the ISO 10993-1 standard (Biological Evaluation of medical devices) using MTT assay. Briefly, from NIH3T3 cells used, 1000 cells were seeded in each sterile hydrogel (6 mm in diameter). Cell groups cultured on a 96-well plate comprised the control group. Then, 3 h after cell attachment, suspensions prepared from each of the CNF and HEC materials were mixed with the concentration used in the hydrogel in the culture medium and poured onto the cells. After 24 and 72 h, the cells were incubated with 100 µL of MTT dye solution (0.5 mg/mL, Sigma-Aldrich, St. Louis, MO, USA, Cat. R8755). After 3 h of incubation, the dye solution was discarded, and the formazan crystals in the cells were dissolved in 100 µL of dimethyl sulfoxide (DMSO). Then, optical density (*OD*) was analyzed using an ELISA plate reader (BioTek) at 230 nm. Finally, Equation (7) was used to evaluate cell survival rate (*P*) in the presence of hydrogels:

(7) $$P\left(\%\right)=\frac{OD_{h}}{OD_{c}}\times 100$$

where, *OD_h_* is the average *OD* for the sample in the presence of a hydrogel, and *OD_C_* is the average *OD* for the control sample.

### 2.4. Statistical Analysis

SPSS statistical software (version 20.0) was used to analyze the data in a completely randomized design. The data are presented as mean ± SD, and the comparison was conducted via the analysis of variance (ANOVA) followed by Tukey’s tests at a 95% confidence level.

## 3. Results

### 3.1. ATR-FTIR

**Figure 2:**
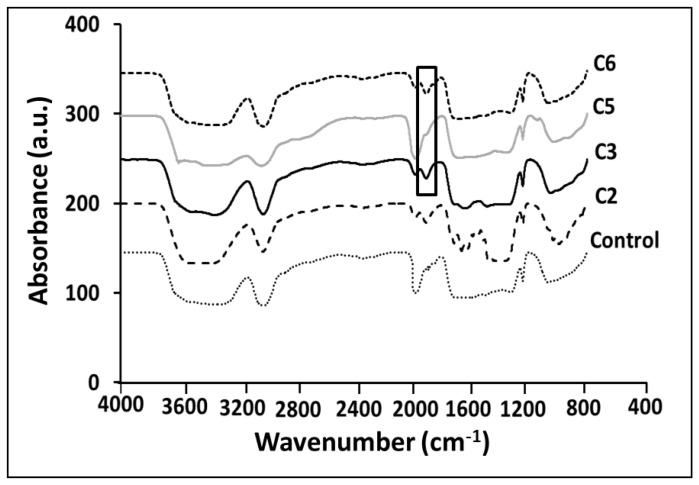
FTIR spectra of the control and group A samples.

Fourier transform infrared (FTIR) spectroscopy was performed to identify the functional groups in the hydrogel samples and the CA-mediated cross-links in the hydrogel samples (Figure 2). Samples C1 and C4 were destroyed in the water absorption test. In other words, they were solved. Therefore, spectroscopy was performed on the remaining prepared samples (C2, C3, C5, and C6). Additionally, a hydrogel made from a mixture of CNF, HEC, and CA cross-linkers in ratios of 10 and 20 wt.%, respectively, without applying heat, was considered as a control sample. The peak observed in the range of 3600 cm^−1^ to 3100 cm^−1^ is related to the stretching vibration of the OH groups [32]. The peak at 1600 cm^−1^ corresponds to the bending of OH groups from adsorbed water [21,33], and the peak present at about 1430 cm^−1^ in the spectra is attributed to the symmetric CH_2_ bending vibration [34,35]. The broad peak at 1100 cm^−1^ indicates stretching of the C–O ether bond [36]. The vibration peak at 890 cm^−1^ is assigned to C–O–C stretching at β-(1-4)-glycosidic linkages. In other words, the glycoside bonds, which are symmetric in polysaccharides, represent the ß-glycoside linkage between glucose units in cellulose [21,36]. In addition, it was observed that the peak in the control sample (a mixture of materials before heating) in the 1715 cm^−1^ region is related to the strong C=O bond in the carboxyl group in CA, and a new peak is observed in the 1738 cm^−1^ and 1740 cm^−1^ regions in the C3 and C6 hydrogel samples, respectively [37]. This may be the result of stretching bond of the carbonyl ester group due to the creation of an ester bond between CNF and CA [37]. Therefore, peak ester formation indicates the proper performance of CA as cross-linking and hydrogel formation, while the absence of this peak in samples C2 and C5 indicates that cross-linking in the lower ratio of CNF to HEC is inadequate, and these samples cannot be used in hydrogel applications.

### 3.2. Gel Fraction

**Figure 3:**
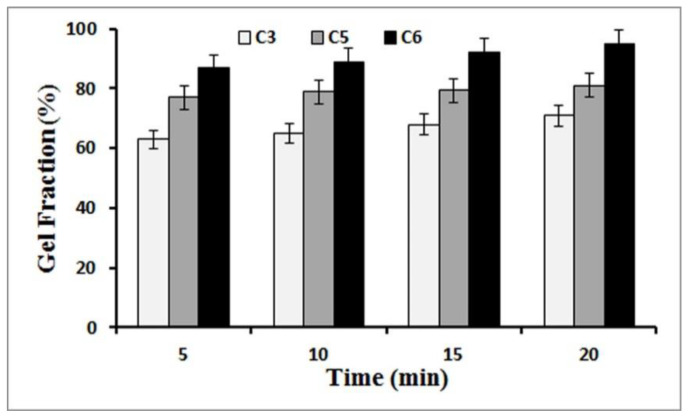
Changes in gel fraction at different times.

Figure 3 shows the gel fraction of the prepared samples, which is an indicator of crosslinking efficiency. It should be noted that samples C1, C2, and C4 were broken immediately after immersion in water. This breakage is probably due to the lower amount of CNF used in these samples. Thus, they were removed from the gel fraction test. As can be seen in Figure 3, the gel fraction increased as the amount of CA cross-linker increased from 10 wt.% to 20 wt.%. The amount of gel fraction of each hydrogel indicated the degree of cross-linking, and an increase in this index is quite normal due to the increase in the amount of the cross-linker. The reason for this could be a gap created between the CNF and the physical barrier, which reduced network formation [38].

### 3.3. Field Emission Scanning Electron Microscopes (FE-SEM)

**Figure 4:**
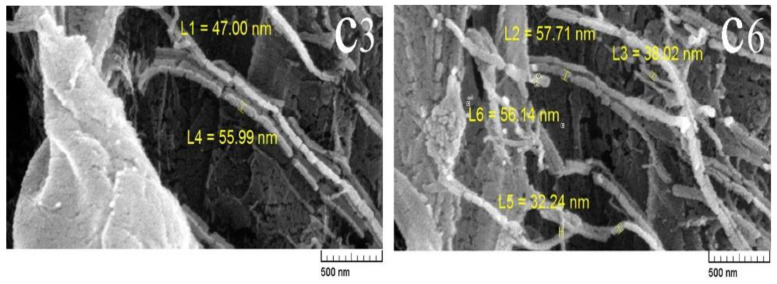
FE-SEM images of C3 and C6 hydrogels.

The morphological properties of the hydrogels, such as the homogeneity of the hydrogel, the dispersion quality of the CNF, and the presence of agglomerated particles, were examined by FE-SEM electron microscopy (Figure 4). The hydrogel produced is composed of tangled microfibrils in a random arrangement without specific orientation or angle. As can be seen, the diameter of the CNF is in the range of 70 to 90 nm. Accordingly, the structure of CNF was preserved after the preparation of the hydrogel, and an accumulation of fibers did not occur during the hydrogel preparation process. It is also observed that the hydrogels have a relatively uniform texture and good CNF dispersion quality, especially in sample C6, as compared to sample C3, which may be due to the use of 20 wt.% CA cross-linkers in sample C6. The CNF in sample C6 have a smaller section than those in C3 due to the higher concentration of CA, which can form more bonds. The preservation of the nano-scale cellulose fibers structure shows that CNF did not agglomerate during the hydrogel preparation process. The presence of HEC in the reaction mixture helped to reduce the accumulation of CNF. Thus, CNF suspended in water has a high tendency for intermolecular reaction and agglomeration [32]. However, the use of HEC creates a gap between the OH groups of CNF, which reduces the intermolecular reactions in CNF [39].

### 3.4. Rheological Properties of Hydrogels

**Figure 5:**
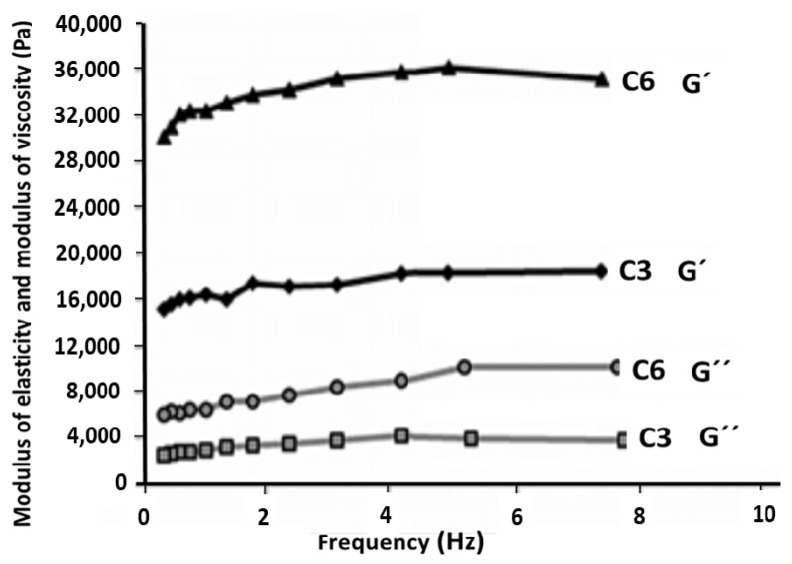
Changes in the elastic and loss modulus of C3 and C6 hydrogels relative to frequency.

The viscoelastic properties and lattice stability of hydrogels were analyzed using rheological studies (Figure 5). Treatments were subjected to variable frequency tests in the range of 0.1 to 8 Hz, in the drawn state. The variable frequency test determines the variable behavior of the viscous and elastic material by changing the frequency. In all treatments, the modulus of elasticity or reserve (G′), as the most sensitive parameter in viscoelastic measurements [40], was higher than the modulus of viscosity or drop (G″), which indicates its dominant behavior. Thus, the fabricated hydrogel is elastic, which was in agreement with the general behavior of all hydrogels with a completely collapsed structure. The value of G’, which was used to estimate the elastic properties, was almost constant and independent of frequency. In addition, the value of G″ was a constant viscosity index, which indicates the formation of a stable network with strong bonds [41,42]. In general, a CNF with good intrinsic properties, such as high surface to volume ratio, flexibility, high OH groups on the surface, and high water-holding capacity, can form gels with high elastic modulus values, which increases the strength of the gel network. Paakko et al. (2007) reported that the modulus of elasticity of CNF suspension is much higher than its modulus of loss [43].

### 3.5. Biocompatibility

Generally, it is very important to study the biocompatibility of hydrogels used in wound dressing and other medical applications [44]. Therefore, the biocompatibility and non-cytotoxicity of the produced hydrogels were evaluated by MTT assay. The results of the MTT test showed that the biocompatibility of the cells in the suspension containing CNF on day 1 and day 3 was 103.6 ± 4.3% and 97.6 ± 1.9%, respectively, and for HEC, it was 91.5 ± 2.8% and 78 ± 4.5%, respectively. Overall, the results of the MTT test confirmed the biocompatibility of the prepared hydrogels. Thus, the experimental results showed no cytotoxicity of the biocompatible hydrogels. The complete similarity of the morphology of cultured cells on the plate with and without the hydrogel also confirms the non-toxicity of the hydrogel.

### 3.6. Drug Release

**Figure 6:**
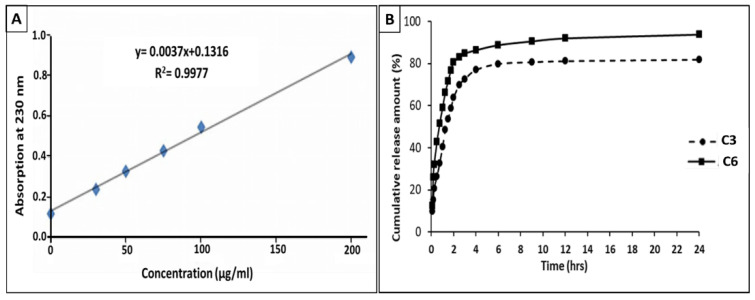
(**A**) Calibration curve of silver nitrate adsorption at 230 nm relative to the concentration and (**B**) Cumulative percentage of silver nitrate release from hydrogels A3 and A6 relative to time.

Figure 6A shows the calibration curve for silver nitrate absorption in PBS in the concentration range of 0 to 200 μg/mL at six points. Light absorption of silver nitrate at 230 nm was measured by ultraviolet/visible spectroscopy (UV-Vis). The results of the drug release analysis showed that there is a linear relationship between the amount of absorption and the concentration of the drug. As can be seen, the fitted regression line was obtained with an R^2^ value greater than 0.99. A value above R^2^ indicates a proper fit of the regression line and the calibration curve [45].

Using the fitting formula, the concentration of each sampled solution from the release medium was obtained, and the cumulative drug release percentage was calculated for both treatments. Figure 6B shows the change of these values with respect to sampling times. The treatments showed an exponential release profile for the cumulative release percentage of silver nitrate from different hydrogels relative to time. A high drug release percentage from 61.6% for C6 treatment to 78.5% for C3 treatment was observed within the first 2 h of drug release, and then the drug was slowly released from the hydrogel for up to 24 h. Total drug release in hydrogels varied from 80.1% in the C6 treatment to 925% in the C3 treatment after 24 h. In a study by Rodriguez et al. (2003), the relatively rapid release of diclofenac sodium, which has a similar solubility to silver nitrate, was reported to be about 70 to 90% in the first 2 h of the test. The drug was then gradually released from the hydrogel derived from the HEC cation [46]. These drug release conditions make hydrogels suitable for use as wound dressings. This is because the system rapidly increases the drug concentration in the burn area, so that the burn reaches the required dose at the beginning of the repair, and the following slower release helps maintain the drug level on the burn area [47]. The gradual release of the drug in the second phase of the experiment, with a final release of 81.8–93.7%, is probably due to the chemical interactions between the OH group of CNF and the positive charge of silver nitrate. By creating a distance between CNF molecules and reducing the intermolecular reaction of OH from CNF, the presence of HEC increases the binding conditions between the drug and the matrix.

### 3.7. Investigation of Drug Release Mechanism

Table 2 shows the linear regression equations fitted to the logarithmic curve with the cumulative percentage of relative release against the logarithm of the time with the regression coefficient R^2^, which indicates the closeness of linear regression to the curve. In studying the mechanism of drug release, the regression coefficients of 0.99 confirm that a suitable line is fitted to each of the curves, and the Peppas-Ritger model is consistent with our results. In this study, the exponential coefficients (*n*) obtained from the linear regression slope are in the range of 0.51 and 0.53 from plate hydrogels prepared from two different types of cross-linkers. Accordingly, the release kinetics include both diffusion and delamination processes. Given that *n* values are closer to the emission range (*n* = 0.5), the predominant mechanism in this release study is diffusion (Table 2). Since the concentration gradient is the driving force in drug release, the concentration difference between the environment and the hydrogel is probably responsible for directing drug release [48]. Therefore, the quasi-experimental Peppas-Ritger equation used to model the drug release from the hydrogel showed that the drug release was affected by two factors: Fick’s release (Higuchi model) and swelling. Our results showed the possibility of hydrogel swelling, in contact with wound secretions, is low due to the use of a semi-wet hydrogel dressing with a humidity of about 70%. In addition, the results of the release modeling assigned a small share to the mechanism of release through swelling and introduced Fick diffusion as the predominant release mechanism.

## 4. Conclusions

According to the FE-SEM image, the structure of the CNF was preserved after the preparation of the hydrogel; minimal fiber agglomeration occurred during the hydrogel preparation process, but the distribution of the components was uniform. The hydrogels were loaded with silver nitrate disinfectant to create burn-healing properties in a burn dressing. The release of the drug was very rapid in the first 2 h, providing the required dose at the beginning of the repair, after which the release was slower and more gradual for the next 24 h, helping to maintain the drug on the surface of the burn. These drug release conditions are suitable for using the hydrogels as a burn dressing. Thus, hydrogels burn dressing used for drug delivery and gradual release of the drug reduces the dose of the drug, which reduces its side effects and prevents the need for rapid and frequent changes of the dressing. The results of release modelling using the Peppas-Ritger quasi-experimental equation made a small contribution to the controlled release mechanism and introduced Fick’s diffusion as the dominant release mechanism. Among the prepared samples, hydrogels with a ratio of 3:1 between NFC and HEC have better strength properties and cross-linking efficiency, and due to their suitable rheological properties and non-cytotoxicity, the potentiality to be used as a burn dressing. In addition to having ideal properties for use in dressings, they also have antimicrobial and pain-relieving burn repair properties when loaded with silver nitrate disinfectant. The developed hydrogels have a dual benefit: on the one hand, they cause a rapid drug concentration in the wound area due to the immediate release. On the other hand, attributed to their antibacterial properties, they repair the burn and relieve pain. In summary, biodegradable hydrogel constructed with CNF containing silver nitrate can be used as a suitable and practical product for burn repair.

## Figures and Tables

**Table 1:** Sample denotation.

Groups	CNF Concentration (wt.%)	CA Concentration (wt.%)	HEC Concentration (wt.%)
C1	1	10	1
C2	2	10	1
C3	3	10	1
C4	1	20	1
C5	2	20	1
C6	3	20	1

**Table 2:** Linear equations, regression coefficients and exponential coefficient obtained from the Peppas-Ritger model of the hydrogels.

Sample	Linear Formula	Diffusion Coefficient (*n*)	Regression Coefficient (R^2^)
C3	y = 0.5294X + 1.7899	0.5394	0.9994
C6	y = 0.5352X + 1.6911	0.5171	0.9966

## Data Availability

Not applicable.

## References

[1] Sood A., Granick M.S., Tomaselli N.L. (2014). Wound dressings and comparative effectiveness data. Adv. Wound Care.

[2] Bharskar G. (2020). A review on hydrogel. World J. Pharm. Pharm. Sci..

[3] Gibas I., Janik H. (2010). Review: Synthetic polymer hydrogels for biomedical application. Chem. Chem. Technol..

[4] Dhivya S., Padma V.V., Santhini E. (2015). Wound dressings–A review. BioMed.

[5] Rivera A.E., Spencer J.M. (2007). Clinical aspects of full-thickness wound healing. Clin. Dermatol..

[6] Strecker-McGraw M.K., Jones T.R., Baer D.G. (2007). Soft tissue wounds and principles of healing. Emerg. Medic. Clinic. N. Am..

[7] Boateng J.S., Matthews K.H., Stevens H.N.E., Eccleston G.M. (2008). Wound healing dressings and drug delivery systems: A review. J. Pharm. Sci..

[8] Kamoun E.A., Kenawy E.-R.S., Chen X. (2017). A review on polymeric hydrogel membranes for wound dressing applications: PVA-based hydrogel dressings. J. Adv. Res..

[9] Xu W., Liu K., Li T., Zhang W., Dong Y., Lv J., Wang W., Sun J., Li M., Wang M. (2019). An in situ hydrogel based on carboxymethyl chitosan and sodium alginate dialdehyde for corneal wound healing after alkali burn. J. Biomed. Mater. Res. Part A.

[10] Naves L.B., Almedia L. (2015). Wound Healing Dressing and Some Composites Such as Zeolite, TiO_2_, Chitosan and PLGA: A Review. Int. J. Metall. Mater. Eng..

[11] Liu R., Dai L., Si C., Zeng Z. (2018). Antibacterial and hemostatic hydrogel via nanocomposite from cellulose nanofibers. Carbohydr. Polym..

[12] Dargaville T.R., Farrugia B.L., Broadbent J.A., Pace S., Upton Z., Voelcker N.H. (2013). Sensors and imaging for wound healing: A review. Biosens. Bioelectron..

[13] Alven S., Nqoro X., Aderibigbe B.A. (2020). Polymer-based materials loaded with curcumin for wound healing applications. Polymers.

[14] Farina J.A., Rosique M.J., Rosique R.G. (2013). Curbing inflammation in burn patients. Int. J. Inflamm..

[15] Sannino A., Demitri C., Madaghiele M. (2009). Biodegradable cellulose-based hydrogels: Design and applications. Materials.

[16] Lu T., Li Q., Chen W., Yu H. (2014). Composite aerogels based on dialdehyde nanocellulose and collagen for potential applications as wound dressing and tissue engineering scaffold. Compos. Sci. Technol..

[17] Sell S.A., Wolfe P.S., Garg K., McCool J.M., Rodriguez I.A., Bowlin G.L. (2010). The use of natural polymers in tissue engineering: A focus on electrospun extracellular matrix analogues. Polymers.

[18] Babu R.P., O’connor K., Seeram R. (2013). Current progress on bio-based polymers and their future trends. Prog. Biomater..

[19] Jiang X., Bai Y., Chen X., Liu W. (2020). A review on raw materials, commercial production and properties of lyocell fiber. J. Bioresour. Manag..

[20] Joseph B., Sagarika V.K., Sabu C., Kalarikkal N., Thomas S. (2020). Cellulose nanocomposites: Fabrication and biomedical applications. J. Bioresour. Bioprod..

[21] Khanjanzadeh H., Behrooz R., Bahramifar N., Gindl-Altmutter W., Bacher M., Edler M., Griesser T. (2018). Surface chemical functionalization of cellulose nanocrystals by 3-aminopropyltriethoxysilane. Intern. J. Bio. Macro..

[22] Sepahvand S., Jonoobi M., Ashori A., Gauvin F., Brouwers H.J.H., Oksman K., Yu Q. (2020). A promising process to modify cellulose nanofibers for carbon dioxide (CO_2_) adsorption. Carbohydr. Polym..

[23] Sepahvand S., Bahmani M., Ashori A., Pirayesh H., Yu Q., Dafchahi M.N. (2021). Preparation and characterization of air nanofilters based on cellulose nanofibers. Int. J. Biol. Macromol..

[24] Moritz S., Wiegand C., Wesarg F., Hessler N., Müller F.A., Kralisch D., Hipler U., Fischer D. (2014). Active wound dressings based on bacterial nanocellulose as drug delivery system for octenidine. Int. J. Pharm..

[25] Xia X., Afshar A., Yang H., Portela C.M., Kochmann D.M., Di Leo C.V., Greer J.R. (2019). Electrochemically reconfigurable architected materials. Nature.

[26] Abazari M.F., Gholizadeh S., Karizi S.Z., Birgani N.H., Abazari D., Paknia S., Derakhshandeh H., Allahyari Z., Amini S.M., Hamidi M. (2021). Recent Advances in Cellulose-Based Structures as the Wound-Healing Biomaterials: A Clinically Oriented Review. Appl. Sci..

[27] Li S.-M., Jia N., Ma M.-G., Zhang Z., Liu Q.-H., Sun R.-C. (2011). Cellulose–silver nanocomposites: Microwave-assisted synthesis, characterization, their thermal stability, and antimicrobial property. Carbohydr. Polym..

[28] Yang T., Long H., Malkoch M., Gamstedt E.K., Berglund L., Hult A. (2011). Characterization of well-defined poly(ethylene glycol) hydrogels prepared by Thiol-ene chemistry. J. Polym. Sci. Part A Polym. Chem..

[29] Trovatti E., Freire C.S.R., Pinto P.C., Almeida I.F., Costa P., Silvestre A.J.D., Neto C.P., Rosado C. (2012). Bacterial cellulose membranes applied in topical and transdermal delivery of lidocaine hydrochloride and ibuprofen: In vitro diffusion studies. Int. J. Pharm..

[30] Qi X., Li J., Wei W., Zuo G., Su T., Pan X., Zhang J., Dong W. (2017). Cationic Salecan-based hydrogels for release of 5- fluorouracil. RSC Adv..

[31] Ritger P.L., Peppas N.A. (1987). A simple equation for description of solute release II. Fickian & Anomalous release from swellable devices. J. Control. Release.

[32] Robles E., Urruzola I., Labidi J., Serrano L. (2015). Surface-modified nano-cellulose as reinforcement in poly (lactic acid) to conform new composites. Ind. Crops Prod..

[33] Barique M.A., Tsuchida E., Ohira A., Tashiro K. (2018). Effect of elevated temperatures on the states of water and their correlation with the proton conductivity of Nafion. ACS Omega.

[34] Ciolacu D., Ciolacu F., Popa V.I. (2011). Amorphous cellulose-structure and characterization. Cellul. Chem. Technol..

[35] Hokkanen S., Bhatnagar A., Repo E., Lou S., Sillanpää M. (2016). Calcium hydroxyapatite microfibrillated cellulose composite as a potential adsorbent for the removal of Cr (VI) from aqueous solution. Chem. Eng. J..

[36] Mulyadi A., Deng Y. (2016). Surface modification of cellulose nanofibrils by maleated styrene block copolymer and their composite reinforcement application. Cellulose.

[37] Abdel-Mohsen S.A., El-Emary T.I. (2018). New pyrazolo [3, 4-b] pyridines: Synthesis and antimicrobial Activity. Der Pharma Chem..

[38] Mohsen M., Gomaa E., Mazaid N.A. (2017). Mohammed, Synthesis and characterization of organic montmorillonite-polyvinyl alcohol-co-polyacrylic nanocomposite hydrogel for heavy metal uptake in water. AIMS Mater. Sci..

[39] García-Astrain C., González K., Gurrea T., Guaresti O., Algar I., Eceiza A., Gabilondo N. (2016). Maleimide-Grafted Cellulose Nanocrystals as Cross-Linkers for Bionanocomposite Hydrogels. Carbohydr. Polym..

[40] Rudraraju V.S., Wyandt C.M. (2005). Rheological characterization of Microcrystalline Cellulose/Sodiumcarboxymethyl cellulose hydrogels using a controlled stress rheometer: Part I. Int. J. Pharm..

[41] Khondkar D., Tester R.F., Hudson N., Karkalas J., Morrow J. (2007). Rheological behaviour of uncross-linked and cross-linked gelatinised waxy maize starch with pectin gels. Food Hydrocoll..

[42] Shimojo A.A., Pires A., Lichy R., Santana M.H. (2015). The performance of crosslinking with divinyl sulfone as controlled by the interplay between the chemical modification and conformation of hyaluronic acid. J. Braz. Chem. Soc..

[43] Paakko M., Ankerfors M., Kosonen H., Nykanen A., Ahola S., Osterberg M., Ruokolainen J., Laine J., Larsson P.T., Ikkala O.T. (2007). Lindstrom, Enzymatic hydrolysis combined with mechanical shearing and high-pressure homogenization for nanoscale cellulose fibrils and strong gels. Biomacromolecules.

[44] Shetye S.P., Godbole A., Bhilegaokar S., Gajare P. (2015). Hydrogels: Introduction, preparation, characterization and applications. IJRM Hum..

[45] Behnia N., Pirouzfar V. (2018). Effect of operating pressure and pyrolysis conditions on the performance of carbon membranes for CO_2_/CH_4_ and O_2_/N_2_ separation derived from polybenzimidazole/Matrimid and UIP-S precursor blends. Polym. Bull..

[46] Rodríguez R., Alvarez-Lorenzo C., Concheiro A. (2003). Cationic cellulose hydrogels: Kinetics of the cross-linking process and characterization as pH-/ion-sensitive drug delivery systems. J. Control. Release.

[47] Wilhelms T.A., Schulze D., Alupeil C.I., Rohrer C., Abel M., Wiegand C., Hipler U.C. Release of polyhexamethylene biguanide hydrochloride (PHMB) from a hydroballanced cellulose wound dressing with PHMB. Proceedings of the 17th Conference of the European Wound Management Association.

[48] Amin M.C.I.M., Ahmad N., Halib N., Ahmad I. (2012). Synthesis and characterization of thermo-and pH-responsive bacterial cellulose/acrylic acid hydrogels for drug delivery. Carbohydr. Polym..

